# Symptoms of common mental disorders and suicidality among female survivors of war related sexual and gender based violence in one stop centers of the Amhara region, Ethiopia: a multicenter cross-sectional study

**DOI:** 10.3389/fpsyt.2025.1456909

**Published:** 2025-02-14

**Authors:** Tsion Michael, Solomon Moges Demeke

**Affiliations:** ^1^ Entrepreneurship Development Institution, Addis Ababa, Ethiopia; ^2^ Department of Psychiatry, College of Health Sciences, Woldia University, Woldia, Ethiopia

**Keywords:** common mental disorders, suicidality, sexual and gender based violence, Amhara, Ethiopa

## Abstract

**Introduction:**

Common mental disorders (CMDs) and suicidality are two of the most common psychological and mental health issues associated with acute and chronic sexual and gender-based violence (SGBV). Thus, the purpose of this study was to determine the magnitude of symptoms of CMDs, and suicidality among females experienced SGBV in Ethiopia.

**Method:**

A cross-sectional study was conducted among 407 female survivors of SGBV in the One Stop Centers of the Amhara region. Data analysis was performed using SPSS version 25. The odds ratio at a p-value of 0.05 was used to determine the strength of the association of the independent variables with CMDs and suicidality.

**Results:**

A total of 407 women participated in the study. Suicidality was reported by a quarter of the survivors (24.1%), while CMDs were reported by nearly two-thirds (61.7%). Being widowed (AOR = 3.0, 95% CI = 3.0 [1.22, 7.66]), having a family history of mental illnesses (AOR = 7.1, 95% CI = 7.1 [4.07, 12.39)], being low-income (AOR = 2.8, 95% CI = 2.8 [1.64, 5.06]), and current drug use (AOR = 2.9, 95% CI = 2.9 [1.63, 5.16]) were all linked with CMDs. Having a history of abortion (AOR = 4.1, 95% CI = 4.1 [1.9, 8.5]), CMDs (AOR = 4.6, 95% CI = 4.6 [2.0, 10.74]), and history of suicide (AOR = 3.41, 95% CI = 3.41 [1.22, 9.55]) were some of the characteristics that were substantially linked with suicidality.

**Conclusion:**

Females with SGBV had a high prevalence of CMDs and suicidality and calls for comprehensive remedies.

## Introduction

Women residing in areas affected by conflict often endure widespread atrocities such as massacres, bombings, and the destruction of their homes. They also suffer injuries and fatalities, leading to displacement, ongoing insecurity, and poverty (1). Women are particularly susceptible to violence and hardships during times of war that are directly linked to their gender. For instance, sexual violence and rape are frequently employed as tactics in warfare in various global conflicts (2, 3).

SGBV is any assault, physical or verbal compulsion, or life-threatening deprivation aimed at a specific woman or girl that results in physical or psychological pain, misery, or the arbitrarily deprived of one’s freedom and fosters female subordination (4, 5). Sexual violence has the potential to inflict severe harm on the mental well-being of the victim, leading to serious impacts in the short, medium, or long term (6). Women are at risk of being kidnapped and forced into slavery, often subjected to torture and sexual abuse (7).

In areas affected by conflict, women and girls are at a higher risk of gender-based violence, which not only results in negative health effects but also hinders their ability to find work and participate in efforts to rebuild (8). During periods of conflict and displacement, women frequently bear a disproportionate burden of sexual violence and exploitation (9, 10). Research has shown that there are alarming rates of sexual violence in areas affected by conflict, with numbers ranging from 2.6% in Ukraine to 21.3% in South Sudan. Terribly, acts of sexual violence and human rights violations related to war persist worldwide, especially during conflicts in both low-income and some high-income nations (2, 11–16).

Gender-based violence (GBV) can have severe consequences on individuals’ physical and mental well-being, resulting in unplanned pregnancies, sexually transmitted infections, and fostering discrimination, stigma, and social isolation within families and communities. It is essential to address GBV and provide assistance to survivors to prevent these negative effects and facilitate healing and recovery (13, 17). There is a clear link between violence and mental health issues among women in research conducted in various countries with different income levels (18–21). The combination of war-related and gender-based violence is likely to have a detrimental impact on the health of women affected by conflict (22, 23). The impact of sexual violence during times of war can have severe psychological effects on survivors. These effects may manifest as post-traumatic stress disorder, anxiety disorders, physical symptoms with no apparent medical cause, psychological pain, dissociative disorders, severe depression, self-harming behaviors including suicidal thoughts, substance abuse, and a distorted perception of oneself and the world (19, 24–26). Gender-based violence is a significant factor in triggering mental health issues in women, stemming from a traumatic experience (27, 28). Females who have encountered gender-based violence are at a substantially greater likelihood of experiencing mental health problems within one to five years following the traumatic incident (29). A study conducted in Australia found that females who have been victims of GBV are twice as probable (58%) to develop typical mental illnesses like depression, anxiety, PTSD, substance abuse, or attempted suicide when compared to females who have not faced GBV (25).

The magnitude of gender-based violence among women in Ethiopia during wartime is alarmingly high, significantly linked to common mental disorders and suicidal behaviors. A study in North Shewa found a GBV prevalence of 58%, with psychological violence at 55%, physical violence at 30.1%, and sexual violence at 16% (30). In Tigray, 43.3% of women experienced at least one type of GBV, with 9.7% reporting sexual violence and 28.6% physical violence (31). Survivors often face mental health challenges, including anxiety (38.6%), depression (27.5%), and post-traumatic stress disorder (12.1%) (31). The SGBV often contributes to suicidal behaviors, with many survivors expressing feelings of hopelessness and isolation (32). A meta-analysis revealed that approximately 46.93% of women experience lifetime VAW, with 37.02% facing violence in the past year (33).

Sexual violence during times of conflict is a serious concern that frequently goes unnoticed or inadequately documented because of a variety of reasons. These may include the protracted duration of conflicts, societal and cultural obstacles, as well as other intricate factors (34). However, to the best of our knowledge no research has been conducted on the association of SGBV with common mental disorders and suicidal behaviors during times of war in Ethiopia. This study aimed to investigate the magnitude of symptoms of common mental disorders and suicidality among women SGBV survivors, as well as the factors associated with these issues. The results of this study will serve as a foundation to understand the extent of potential mental health consequences among GBV survivors in the Amhara Region of Ethiopia during periods of conflict. Additionally, the findings will offer valuable insights for humanitarian organizations, national and local authorities, in order to provide comprehensive support for survivors and to reduce the burden of the mental health consequences of SGBV.

## Methods

### Study design, setting, and participants

An institutional based cross-sectional study was conducted from December/2022 to March/2023, focusing on survivors of gender-based violence in the Amhara Region. The Amhara region of Ethiopia has a vibrant historical and cultural background shaped by its ethnic identity and socio-political dynamics. Known for its diverse cultural heritage, it offers numerous attractions that entice tourists globally (35). The region has been deeply impacted by war-related sexual violence, especially during the recent conflict involving the Tigray People’s Liberation Front. These acts have resulted in severe psychological and physical consequences for survivors. A total of 407 women who had sought assistance from the One Stop Centers in Debre Birhan, Dessie, and Woldia were included in the research. One-Stop Centers (OSCs) are facilities that offer an integrated, multi-sectoral response to the needs of survivors of SGBV in a single location. This facilitates access to essential services during a traumatic period and helps reduce trauma by minimizing the number of providers involved. As a result, survivors can avoid the distress of repeatedly recounting their experiences (36). These centers are designed to provide integrated services to survivors, ensuring access to medical, psychological, and legal support all in one location. They provide free services, including medical evaluations, treatment, psychosocial support, and legal aid. The center is staffed by a dedicated team that includes general practitioners, midwives, nurses, psychologists, pharmacists, laboratory professionals, and legal advisors (37). These centers were chosen from three different zones within the region. The participants were selected using a purposive sampling method, after the sample size proportionally allocated to each center.

Individuals 18 years and older were eligible to participate in the research study. The data were gathered by three local female professionals who are experts in psychology and social work, working at the One Stop Centers. Participants who were seriously ill, mentally incapacitated, or physically disabled were not included in the study. The Institutional Review Board of Addis Ababa University approved all procedures of the study, and ethical approval was obtained from each study site. Additionally, a support letter was obtained from the administrator of each study site. Prior to the interviews, verbal consent was obtained from all participants. They were informed that participation was voluntary and they had the right to withdraw at any time. Confidentiality of the information provided was guaranteed. Participants were also informed about their right to decline participation or withdraw at any time. Those who screened positive during the data collection process were referred to mental health services at the Hospital for further evaluation. The study followed the STROBE (Strengthening the Reporting of Observational Studies in Epidemiology) reporting guidelines.

### Data collection instruments

Data was collected using a structured interviewer administered questionnaire to gather information on socio-demographic details, stress factors, the level of social support, the presence of medical conditions and a family background of mental disorders, as well as past or present use of substances for non-medical purposes.

### Sociodemographic information

The first part of the structured interview comprised questions assessing sociodemographic characteristics of the participants. This included age, marital status, religion, residence, educational level, participant occupational status, partner’s educational level, spouse’s occupational status, and income.

### Mental health related conditions

Symptoms of CMDs were assessed using the 20-item Self-Reporting Questionnaire (SRQ-20). SRQ has 20 items that indicate symptoms of non-psychotic mental illnesses. The SRQ-20 has a high internal consistency reliability (0.78), a sensitivity of 78.6%, and a specificity of 81.5% (38). Participants were taken to have CMDs in the previous month if their score was above or equal to eight (39).

#### Suicidality

the three forms of suicidal behaviors include suicidal ideation, planning, and attempt. The revised suicidal behavior questionnaire (SBQ-R) was used to assess suicidal behaviors. It has an overall result ranges from 3-18; participants who scored greater than or equal to seven were indicating suicidal behaviors (40).

#### Substance use

the first two items of WHO ASSIST (Alcohol, Smoking, and Substance Involvement Screening Test) were employed to assess lifetime and present substance use. Current users had used substances (alcohol, khat, cigarettes) in the previous three months. Those who had used substances (alcohol, khat, cigarettes, and others) at least once were considered ever-users (41).

#### Stressful life events

the List of Threatening Experiences (LTE) was used to evaluate stressful situations. It was designed to assess stressful life experiences and includes 12 types of unpleasant life occurrences. Participants were divided into two groups: those who experienced stressful life events of 0, 1, or 2 and those who had at least one or more tough life events in the prior six months (42).

#### Social support

the participants’ social support was assessed using the Oslo social support scale (Oslo-3). According to the Oslo social support scale (Oslo-3), which has a score range of 3 to 14, participants with scores of three to eight were considered to receive poor social support, and those with scores of 9 to 11 were considered to have moderate social support. In contrast, those with scores of between 12 and 14 were considered to have good social support (43).

### Data quality control

The structured questionnaires were prepared in English, translated into Amharic (the local language) and then back translated into English to ensure the reliability and comprehensibility of the instruments by different language experts. The instruments were pretested in 5% (21) of the sample size in Shewa Robit Hospital. The final version of the questionnaires was established following the feedback from the pre-test. Data collectors and supervisors received a training regarding the study instruments and procedures, and ethical issues. The questionnaires were checked for its consistency and understandability. Supervisors carried out supervision at each site. The filled questionnaires were checked for completeness and reliability. Informed consent was obtained from each participant in verbal form prior to assessments.

### Data analysis

At the end of the data collection, the questionnaires were checked for completeness. The data were edited, cleaned, coded, and entered into the computer using Epi-Data 4.6 version and exported, and analyzed using Statistical Package for Social Sciences (SPSS) version 25 (IBM). Analysis was done using both descriptive and inferential statistics. Descriptive statistics were used to describe the findings, including frequencies, and percentages. The presence of an association between symptoms of common mental disorders and suicidality, and independent variables was assessed using logistic regression analysis. Variables significant at bi-variable logistic regression i.e., p < 0.20 were entered into multivariable logistic regression. Statistical significance was considered at a p-value less than 0.05, and the strength of association was estimated in odds ratio with a 95% confidence interval.

## Results

### Descriptive results on participant sociodemographic characteristics among SGBV survivors of Amhara region

The study included a total of 407 participants, with a response rate of one hundred percent. More than half of the respondents, 58%, were Orthodox Christians, 35.6% were Muslims, and 6.4% were Protestants. Below half were married (167 [41%]), while (113 [27.8%]), (85 [20.9%]), (42 [10.3%]), were single, widowed, and divorced or separated respectively. Three fourth of the participants (309 [75.9%]) lived in urban. Most participants (251 [61.7%]) had complete primary school (See **Table 1**).

**Table 1:** Socio demographic characteristics of SGBV survivors of Amhara Region (n=407).

Variables	Frequency (N=407)	Percentage (%)
Age		
18-25 years	168	41.3
26-49 years	197	48.4
>=50 years	42	10.3
Marital status		
Married	167	41.0
Single	113	27.8
Widowed	85	20.9
Divorced and/separated	42	10.3
Religion		
Orthodox Christian	236	58.0
Muslim	145	35.6
Protestant	26	6.4
Residence		
Urban	309	75.9
Rural	98	24.1
Educational level		
Cannot read and write	41	10.1
Primary education	251	61.7
Secondary education	100	24.6
Post-secondary education	15	3.7
Participant Occupational status		
Housewife	143	35.1
Trader	57	14.0
Employee	58	14.3
No job	133	32.7
Other	16	3.9
Partner’s educational level		
Cannot read and write	9	2.2
Primary education	107	26.3
Secondary education	42	10.3
Post-secondary education	5	1.2
Spouse’s Occupational status		
Farmer	44	10.8
Trader	52	12.8
Employee	61	15.0
No job	4	1.0
Other	1	.2
Participant Income		
3052.4 ETB	232	57.0
>3052.4 ETB	175	43.0

### Clinical and substance related characteristics of SGBV survivors of Amhara region (n=407)

More than half of the respondents (50.6%) had relatives with a history of mental illness, and more than three-quarters (79.9%) had suffered physical harm due to the sexual assault. Around 19.4% had unwanted pregnancies, 17% aborted pregnancies, and 2.5% gave birth to unwanted children. Three-fourths (75.2%) of the participants used substances in their lifetime, and 69.3% used them in the past three months (See **Table 2**).

**Table 2:** Clinical and substance related characteristics of SGBV survivors of Amhara Region (n=407).

Variables	Frequency (N=407)	Percentage (%)
Previous mental illnesses		
Yes	4	1.0
No	403	99.0
Mental illnesses within the family		
Yes	206	50.6
No	201	49.4
Major physical diseases		
Yes	173	42.5
No	234	57.5
If ‘yes’ type of medical condition/s		
Hypertension	25	6.1
Diabetes	31	7.6
Epilepsy	14	3.4
HIV AIDS	42	10.3
Sexually transmitted disease	52	12.8
Other	9	2.2
Physical injury due to the assault		
Yes	325	79.9
No	82	20.1
Exposed to unwanted pregnancy		
Yes	79	19.4
No	328	80.6
Abortion following the assault		
Yes	69	17.0
No	338	83.0
Having a baby, they do not want		
Yes	10	2.5
No	397	97.5
Physical pain following the assault		
Yes	185	45.5
No	222	54.5
Lifetime substance use		
yes	306	75.2
no	101	24.8
Alcohol		
Yes	257	63.1
No	150	36.9
Khat		
Yes	94	23.1
No	313	76.9
Tobacco		
Yes	12	2.9
No	395	97.1
Shisha		
Yes	29	7.1
No	378	92.9
Current substance use		
yes	282	69.3
No	125	30.7
Alcohol		
Yes	166	40.8
No	241	59.2
Khat		
Yes	67	16.5
No	340	83.5
Tobacco		
Yes	7	1.7
No	400	98.3
Shisha		
Yes	7	1.7
No	400	98.3
Types of SGBV		
Physical violence	224	55.1
Sexual violence	101	24.8
Emotional and psychological violence	82	20.1

### Prevalence of symptoms of common mental disorders and suicidality among SGBV survivors of Amhara region (n=407)

**Figure 1:**
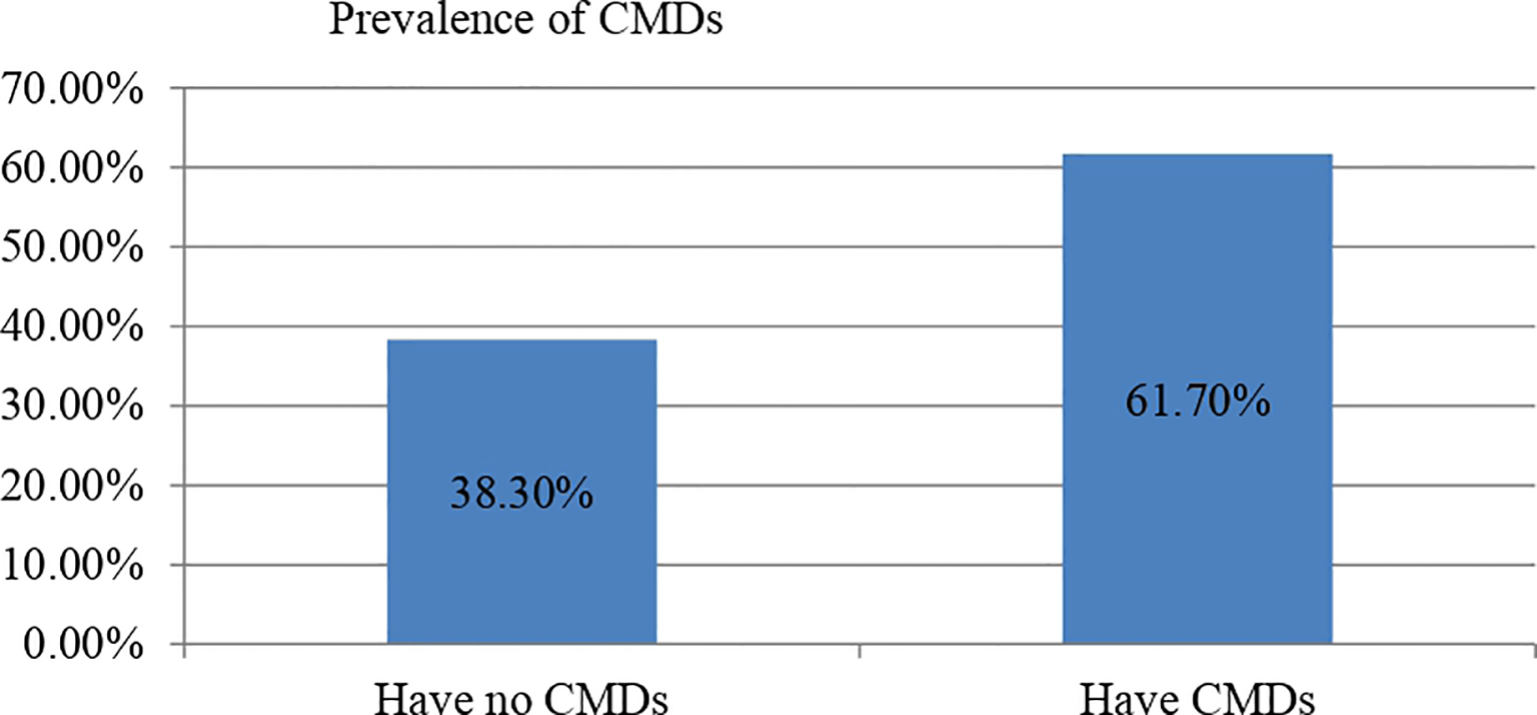
Prevalence of symptoms of common mental disorders among SGBV of Amhara Region, (n = 407).

**Figure 2:**
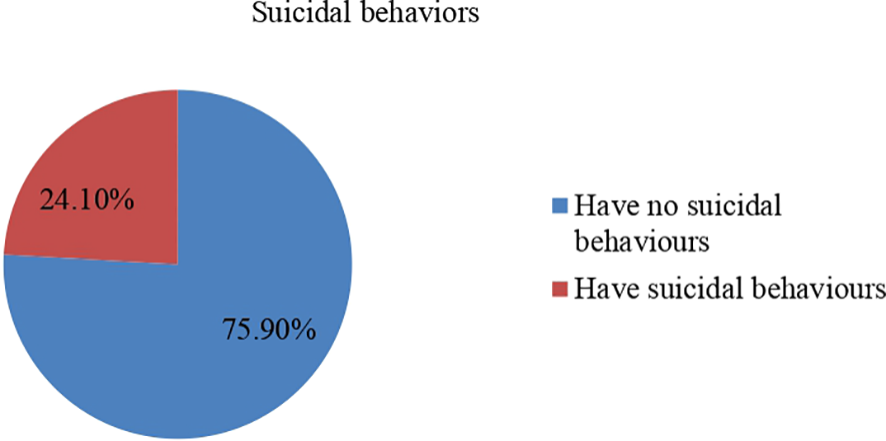
Prevalence of suicidality among SGBV survivors of Amhara Region, (n= 407).

Regarding the specific numbers related to the SGBV experienced by the participants, 224 (55.1%) reported experiencing physical violence, 101 (24.8%) reported sexual violence, and 82 (20.1%) reported emotional and psychological violence. Of those who had SGBV nearly two third (61.7%) of the participants were found to have common mental disorders (95% CI = [56.8% - 66.3%]) whereas 24.1% of the participants reported having suicidal behaviours (95% CI = [19.37% - 28%]) (See **Figures 1**, **2**). Suicidal thought, plan, and attempt rates have been determined as (206 [50.6%], 21 [5.1%], and 68 [16.8%]), respectively.

### Association of SGBV with common mental disorders and suicidal behaviours

The study found the following factors to be significantly associated with common mental disorders: being widowed, having a family history of mental illness, a poor income, experiencing a stressful life event, and substance use in the previous three months. Compared to their counterparts, widowed participants had three times the odds of having common mental disorders (AOR= 3.0, 95% CI= [1.22 to 7.66]). When compared to those who did not have a family history of mental illness, the odds of developing common mental disorders were 7.1 times higher (AOR=7.1, 95% CI; [4.07 to 12.39]). Participants with less than 3052.4 ETB in a month’s earnings were 2.8 times (AOR=2.8, 95% CI= [1.64 to 5.06]) more likely to have common mental disorders than those with more than 3052.4 ETB in income per month. The odds of developing common mental disorders were 2.9 times greater among those who used substances (AOR=2.9, 95% CI= [1.63, 5.16]) than among nonusers. Age and social support were found to be unrelated to CMDs (See **Table 3**).

**Table 3:** Bivariable and Multivariable binary logistic regression analysis of common mental disorders among SGBV survivors of Amhara Region (n=407).

Explanatory variables	Common mental disorders		COR	AOR	P-value
	Yes	No			
Age					
18-25 years	57	111	0.45 (0.19, 1.05)	0.63 (0.22, 1.84)	0.40
26-49 years	91	106	0.27 (0.12, 0.62)	0.44 (0.16, 1.20)	0.11
>=50 years	8	34	Ref.		
Marital status					
Married	76	91	Ref.		
Single	53	60	0.94 (0.58, 1.52)	0.5 (0.27, 1.08)	0.08
Widowed	9	76	7.05 (3.31, 15)	3.0 (1.22, 7.66)	0.01
Divorced/separated	18	24	1.11 (0.56, 2.2)	1.4 (0.60, 3.41)	0.41
Income					
3052.4 ETB	59	173	0.27 (0.18, 0.42)	2.8 (1.64, 5.06)	0.001
>3052.4 ETB	97	78	Ref.		
Family history of mental illnesses					
Yes	31	175	9.285 (5.76, 14.95)	7.1 (4.07, 12.39)	0.001
No	125	76	Ref.		
Stressful life events					
Has no stressful event	47	14	Ref.		
1/2 stressful events	19	34	6.00 (2.64, 13.63)	5.3 (1.9, 14.51)	0.001
>=3 stressful events	90	203	7.57 (3.96, 14.45)	5.6 (2.5, 12.54)	0.001
Current substance use					
Yes	85	197	3.04 (1.97, 4.71)	2.9 (1.63, 5.16)	0.001
No	71	54	Ref.		
Social support					
Poor social support	79	112	0.65 (0.36, 1.16)	0.55 (0.24, 1.22)	0.14
Moderate social support	55	91	0.75 (1.16, 1.38)	0.65 (0.28, 1.52)	0.32
Strong social support	22	48	Ref.		

In contrast, in the multivariable analysis, being single, being widowed, having a mental illness in the family and having chronic physical issues, history of abortion, having physical illnesses related with the violence, those who had CMDs and previous history of suicide were significantly associated with suicidal behaviors. Participants with CMDs had a 4.6 fold (AOR=4.6, 95% CI= [2.0 to10.74]) increased likelihood of suicide behavior than those without CMDs. Survivors with a past suicide history were 3.4 times (AOR= 3.41, 95% CI= [1.22 to 9.55]) more likely to engage in suicidal behavior than their counterparts. When compared with individuals who did not have abortion, the odds of having suicidal behavior were 4.1 times (AOR= 4.1, 95% CI= [1.9 to 8.5]). Age, residence, educational position, income, stressful life events, and relatives with a history of suicide were not significantly associated with suicidal behavior (See **Table 4**). Surprisingly, the variables associated with the types of SGBV did not demonstrate a significant association with CMDs and suicidal behavior in the bivariable logistic regression analysis (See **Table 2**).

**Table 4:** Bivariable and Multivariable binary logistic regression analysis of suicidal behaviours among SGBV survivors of Amhara Region (n=407).

Explanatory factors	Suicidal behaviours		COR	AOR	P-value
	Yes	No			
Age					
18-25 years	134	34	0.17 (0.084, 0.35)	0.18 (0.07, 1.49)	0.211
26-49 years	158	39	0.16 (0.083, 0.34)	0.25 (0.09, 1.64)	0.304
>=50 years	17	25	Ref.		
Marital status					
Married	141	26	Ref.		
Single	87	26	1.62 (0.88, 2.97)	2.56 (1.10, 5.96)	0.02
Widowed	53	32	3.27 (1.78, 6.0)	1.69 (0.72, 3.96)	0.22
Divorced/separated	28	14	2.71 (1.26, 5.83)	3.52 (1.30, 9.47)	0.01
Residence					
Urban	246	63	Ref.		
Rural	63	35	2.16 (1.31, 3.56)	1.45 (0.67, 3.11)	0.33
Educational level					
Unable to read and write	22	19	Ref.		
Primary education	200	51	0.29 (0.14, 0.58)	0.37 (0.13, 1.99)	0.34
Secondary education	78	22	0.32 (0.15,.70)	0.49 (0.15, 1.59)	0.24
College and above	9	6	0.77 (0.23, 2.56)	0.77 (0.15, 3.91)	0.75
Income					
<=3052.4 ETB	167	65	1.67 (1.04, 2.69)	0.73 (0.38, 1.4)	0.34
>3052.4 ETB	142	33	Ref.		
Mental illnesses in the family					
Yes	138	68	2.8 (1.73, 4.56)	1.96 (1.1, 3.82)	0.04
No	171	30	Ref.		
Physical conditions					
Yes	112	61	2.9 (1.81, 4.63)	1.98 (1.07, 3.64)	0.02
No	197	37	Ref.		
History of abortion					
Yes	37	32	3.56 (2.06, 6.14)	4.1 (1.9, 8.5)	0.001
No	272	66	Ref.		
Physical illnesses related with the violence					
Yes	117	68	3.72 (2.28, 6.05)	2.21 (1.17, 4.18)	0.01
No	192	30	Ref.		
Stressful life events					
Has no stressful event	54	7	Ref.		
1/2 stressful events	45	8	1.37 (0.46, 4.07)	1.5 (0.36, 6.13)	0.57
>=3 stressful events	210	83	3.04 (1.33, 6.97)	1.5 (0.52, 4.57)	0.42
CMDs					
Have CMDs	166	85	5.63 (3.01, 10.52)	4.6 (2.0, 10.74)	0.000
Have no CMDs	143	13	Ref.		
Previous history of suicide					
Yes	15	18	4.41 (2.12, 9.13)	3.41 (1.22, 9.55)	0.01
No	294	80	Ref.		
Family history of suicide					
Yes	14	19	5.06 (2.43, 10.55)	2.61 (0.90, 7.58)	0.076
No	295	79	Ref.		

## Discussion

### Prevalence of CMDs and suicidal behaviours

CMDs in GBV survivors in selected One Stop Centers in Amhara Region were 61.7%. The findings are consistent with an earlier Ethiopian study, which found a prevalence rate of 59.6% (44); however, it is higher than the study done in Australia (37%) (29), and lower than the study from Jamaica (77.3%) (25). The possible discrepancy could be attributable to differences in study design and the tool employed to assess the outcome variable. In this study, the SRQ-20 was employed to assess common mental disorders, whereas, in Australia and Jamaica, the Composite International Diagnostic Interview Version 3.0 was employed. The study in Australia employed a retrospective methodology, but the study in Jamaica used a high sample size. Another possible explanation for the disparity in the frequency of common mental disorders among survivors of gender-based violence is that the study was done in a post-war context.

Suicidal behavior was reported by nearly a quarter of the participants (24.1%). This is consistent with the findings of an Australian study, which showed a prevalence of (19.5%) (29). The current study’s prevalence rate, however, is higher than that reported in research conducted in five low and middle-income countries, which is (10.3%) (45), and Ethiopia (9.3%) (40). The possible reason for this variation is that previous research may have been conducted on a community sample and primary care attendees from the general population. Previous research employed the suicidality module of the Composite International Diagnostic Interview to assess suicidal behavior. On the other hand, the current study was conducted in a sample from war-affected areas to measure suicidal behavior using the Suicide Behaviors Questionnaire-Revised.

### Factors associated with suicidal behaviors

This study found a significant association between several independent variables and suicidal behavior. Of these factors, CMDs is one of the crucial factors contributing to suicidal behavior. CMDs were 4.6 times more common in people with Suicidal behavior than those without. Several studies conducted in low- and middle-income nations support this fact (45), Brazil (46), South Africa (47), and Ethiopia (48). All these studies confirmed that suicidal behavior is significantly associated with a common mental disorder. Experiencing physical and psychological trauma affects the survivor’s mental health, leading to suicide (49, 50). This might be due to the serotonergic deficiency and hypo activity associated with psychiatric disorders such as depression which increased the risk of suicide (51, 52). Another probable explanation is that depression directly influences survivors, making them feel frustrated, alone, and worthless, leading to suicidal conduct (53, 54).

The current study found that being single and widowed/separated were significantly related to suicidal behaviors. The odds of developing suicidal behavior were (AOR= 2.56) and (AOR= (3.52) times higher in those who are single and widowed/separated, respectively, than in their counterparts. This is congruent with the findings of studies from world mental health surveys conducted in seventeen countries, which found that being unmarried is one of the factors significantly correlated with suicide behavior (46).

Participants who had abortions had fourfold greater odds of having suicidal behavior than those who had not undergone abortions. This is consistent with a study conducted in the United States (55). This could be because sexual violence increases the likelihood of unwanted pregnancy and the decision to undergo an abortion. Abortion, in turn, may induce a mood disorder in a vulnerable individual. A woman with a mood problem is less likely to carry out a pregnancy, preferring abortion (55). Those with a suicide history were (AOR=3.41) times more likely to be involved in suicidal behaviors than those without a suicidal history. The finding has been supported by research conducted in Tigray, Ethiopia (48) and Washington (56), which reported that previous suicidal behavior is associated with current or recent suicidal behavior.

Those with relatives with a history of mental illness were nearly twice as likely to develop suicidal behaviors as those without a comparative history of mental illness. This is also consistent with research conducted in Tigray, Ethiopia (48). The possible reason may be that the presence of a family history of mental health problems might disturb their social life due to the effect of stigma and discrimination, which ultimately leads to poor social support and further worsen the risk of having suicidal behavior (57, 58).

The odds of developing suicidal behavior were nearly twice as likely (AOR=1.98) among those with chronic illnesses and were (AOR=2.21) times more likely among those with violence-related physical health problems than their counterparts. These findings were consistent with studies conducted in Greece (59), Kenya (60), Canada (61), and a review of literature of studies conducted in Africa (62). The possible explanation for this might be that chronic illnesses are often related to functional limitations, dissatisfaction with their life, and reduced quality of life, which in turn. Due to the restriction to daily living activities, functional limitations cause discouragement, depression, and suicidality (63, 64). In addition, a history of violence could be a risk factor for suicidal behavior because repeated exposure to painful stimuli increases the risk for suicidality (65).

### Factors associated with common mental disorders

The study found that having relatives with a history of mental illness increases the likelihood of having common mental disorders by 7.1 times compared to not having a comparative history of mental illness. This is consistent with the findings of research done by Illu Ababora (66). A possible explanation can be that the mental disorders often exhibit a genetic component, with studies indicating that individuals with a family history of mental illness are more likely to inherit vulnerabilities to these conditions (67). Additionally, experiencing sexual abuse serves as a significant stressor that can lead to profound and long-term psychological distress (68).

A monthly income of less than or equal to 3052.4 ETB is significantly associated with common mental diseases. Those with a monthly income of less than 3052.4 ETB are 2.8 times more likely to suffer from a common mental disorder higher than those with a monthly income greater than 3052.4 ETB. The likely reason for this association is that many of the participants (35.1%) were homemakers and were unemployed (32.7%) and were expected to take care of many household responsibilities may predispose them to be financial, emotional, and physical strain and make them prone to psychological problems (69).

Compared to individuals who do not use substances, the risk of a common mental disorder is approximately three times higher. Current substance usage was associated with an increased chance of developing a common mental disorder. Butajira’s findings are consistent with earlier research (70) and Jimma, Ethiopia (71). The possible reason for this is that individuals with mental distress are likely to use substances to improve their symptoms (71).

The odds of having a common mental disorder among widowed participants have three times the odds of developing a common mental disorder when compared to their counterparts. The result is in line with a study done in Ethiopia (70). The possible explanation for this is that widowed individuals are likely to lose their primary source of emotional and social support, and this will, in turn, lead them to helplessness, hopelessness, isolation, and depression (72, 73).

The odds of developing common mental disorders are (AOR=5.3) times greater in individuals with one or two stressful life experiences and (AOR=5.6) times higher in those with three or more stressful life events. The result is supported by earlier research done in (74), Iran (75), and Brazil (76). This finding can be explained by the fact that stressors may alter the body’s reaction by firing the sympathetic nervous system and the hypothalamic-pituitary-adrenal axis. Stress causes a rise in oxidative stress, contributing to the deterioration of cells (77, 78). Furthermore, stressful life experiences may play a unique role in the start and worsening of psychiatric issues (79, 80).

## Conclusions

The current study showed an increased prevalence of common mental disorders and suicidal behavior among survivors of gender-based violence. Common mental disorders are substantially associated with being widowed, having relatives with a history of mental illness, having a low income, having life stresses, and using substances in the past three months. Being single and widowed, having relatives with a history of mental illness, developing a chronic medical condition, developing a history of abortion, having a physical illness related to violence, CMDs, and having a previous history of suicide were the factors significantly associated with suicidal behavior. The high prevalence rates necessitate comprehensive interventions such as screening and treatment of common mental disorders and suicide among survivors of gender-based violence.

### Strengths and limitations of the study

This study has strengths and limitations. Standard instruments were used to measure the outcome variables. Health professionals who are experienced were collected the data. The research will also serve as a baseline for further studies. However, the study has limitations. Its cross-sectional design restricts the ability to establish causality between risk factors and mental health outcomes. In addition, selecting participants solely from help centers may introduce selection bias, as women seeking assistance may differ in characteristics and levels of CMDs and suicidality from those who do not. This limits the generalizability of the results to the broader affected population and makes it challenging to apply findings to similar contexts in Ethiopia or elsewhere. To enhance the representativeness of the sample, future studies should include women from various contexts, both urban and rural, as well as those not seeking help at centers. Furthermore, it is important to explore cultural and stigma-related factors that could influence women’s access to mental health support and alter the relationship between risk factors and CMDs or suicidality.

## Data Availability

The raw data supporting the conclusions of this article will be made available by the authors, without undue reservation.
